# Correlation between tear film lipid layer thickness and transepidermal water loss from the ocular area in patients with dry eye disease and in healthy controls

**DOI:** 10.1371/journal.pone.0270810

**Published:** 2022-07-20

**Authors:** Do Yeh Yoon, Jeon Hee Eun, Joon Young Hyon

**Affiliations:** 1 Department of Ophthalmology, Seoul National University College of Medicine, Seoul, Korea; 2 Department of Ophthalmology, Seoul National University Bundang Hospital, Seongnam, Korea; Saarland University, GERMANY

## Abstract

**Purpose:**

This study aimed to evaluate the correlation between tear film lipid layer thickness and transepidermal water loss (TEWL) from the ocular area in patients with short tear break-up time (TBUT)-type dry eye and healthy controls.

**Methods:**

This prospective study included 25 eyes of patients with short TBUT-type dry eye disease and 25 eyes of healthy controls.

**Results:**

Tear film lipid layer thickness was measured using an interferometer, and TEWL from the ocular area was measured using a Tewameter TM300 with custom-made goggles. The correlation between tear film lipid layer thickness and TEWL was evaluated. Additionally, other parameters such as TBUT, Schirmer I score, ocular surface staining, the presence and type of meibomian gland dysfunction (MGD), ocular surface disease index (OSDI), and visual analog scale (VAS) scores were evaluated. Tear film lipid layer thickness did not show a significant correlation with TEWL from the ocular area measured using a Tewameter TM300. However, tear film lipid layer thickness was significantly correlated with tear break-up time (p = 0.004) and ocular surface staining by NEI (National Eye Institute) scheme (p = 0.03). TEWL showed positive correlation with the Schirmer I score *(*p = 0.004).

**Conclusions:**

The tear film lipid layer affected the stability of the tear film more than the amount of TEWL in patients with short TBUT dry eye and healthy controls.

## Introduction

Dry eye disease (DED) is a chronic multifactorial disease of the ocular surface and is one of the most common ocular diseases. The TFOS Dry Eye Workshop II (TFOS DEWS II) defined DED as an ocular surface disease that is characterized by loss of homeostasis of the tear film and ocular symptoms, including tear film instability and hyperosmolarity, ocular surface inflammation, and ocular surface damage [1]. DED is classified into two etiological subtypes: aqueous deficiency (ADDE) and evaporative (EDE); however, clinically, there is frequent co-existence and overlap of the subtypes. This study included patients with short TBUT dry eye and healthy controls. Short TBUT type dry eye is characterized by 1) a TBUT of less than 5 seconds with minimal vital staining, 2) normal tear production, and 3) dry eye symptoms such as ocular fatigue or dryness. The significance of this type of dry eye has recently been gaining prominence [2]. We chose this subtype of DED to minimize the effects of a compromised ocular surface or severe desiccation on the results.

Excessive tear evaporation and the lack of tear production, which leads to tear hyperosmolarity and results in ocular surface inflammation, is considered a key factor that induces a vicious cycle in the pathogenesis of DED [1].

In a three-layered model of tear film, a tear film lipid layer has been recognized as a barrier to tear evaporation [3, 4]. Recent advances in technology using interferometry can measure the lipid layer thickness (LLT) in tear film. Tear evaporation was measured by measuring the change in relative humidity in a conditioned chamber or ventilation chamber or by calculating the change in tear volume from thermodata obtained using infrared thermography [5–8]. In a previous study, we measured transepidermal water loss (TEWL) from the ocular area using Tewameter TM300 in moderate to severe DED cases and showed that TEWL may reflect tear evaporation [9].

This study aimed to investigate the correlation between LLT, TEWL, and other DED parameters, including the status of meibomian gland dysfunction (MGD). We hypothesized that tear film LLT can affect TEWL from the ocular area.

## Materials and methods

### Participants

This single-center, prospective case-control study included 25 eyes of 25 patients with mild DED (Dry Eye Workshop dry eye severity level ≤1) [10] and 25 eyes of 25 age-matched healthy subjects as controls. The study was conducted in accordance with the tenets of the Declaration of Helsinki, and the study protocol was approved by the Institutional Review Board of Seoul National University Bundang Hospital (no. B-1807/483-104). Data of dry eye patients who fulfilled the following diagnostic criteria were collected: age of 20 years or older; presence of ocular symptoms and tear break-up time (TBUT) less than 5 seconds; dry eye severity level ≤1 according to the Dry Eye Workshop grading system [10]; absence of history of ocular surgery, injury, and allergy; and no use of anything other than non-preserved artificial tears. Exclusion criteria included Sjogren’s syndrome, contact lens use, systemic disease or medication use that may cause DED, skin disease that may affect evaporation, and an MGD severity level ≥4 [11]. Moreover, data of healthy subjects who fulfilled the followed diagnostic criteria were collected: age of 20 years or older; absence of ocular symptoms; TBUT 5 seconds or higher; absence of history of ocular surgery, injury, and allergy; and no use of anything other than non-preserved artificial tears. The exclusion criteria was same as those of the study group. Potential participants were screened for eligibility, and written informed consent was obtained from each participant by an investigator after explanation of the nature and possible consequences of the study.

Tear collection and measuring tear osmolarity (TearLab) were performed separately. TEWL was measured 30 minutes after the slit lamp examination, and Tearlab was performed 30 minutes later. Afterward, tear collection and Schirmer test were performed at 10-minute intervals.

### TEWL measurement

The Tewameter^®^ TM 300 (Courage & Khazaka Electronic GmbH, Cologne, Germany) measures the density gradient of water evaporation from the skin indirectly using the two pairs of sensors (temperature and relative humidity) inside the hollow cylinder [9]. Using a customized goggle-like adapter cap, this device can be used to measure TEWL from the ocular surface and periorbital skin [9]. The measurements were performed in a test room equipped with a thermo-hygrostat (room temperature, 24–26°C; relative humidity, 35–45%). After the participant sat in the room for a 10-minute equilibration period, an experienced investigator (HEJ) measured the TEWL (measured unit g/hm^2/s; hm: hectometer) at 1-second intervals until the values reached a plateau. The average of five values and the time taken to reach a plateau were used for further analyses. The participants were instructed to look forward with their eyes blinking normally during the recording.

### Dry eye parameters

The dry eye parameters that were obtained included TBUT, ocular staining scores (OSS), Schirmer-I test values, the ocular surface disease index (OSDI), visual analogue scale (VAS) scores as symptom scores, tear osmolarity, and tear matrix metallopeptidase 9 (MMP-9). All parameters were assessed in the same test room as described above. To obtain the TBUT measurements and ocular staining score, we stained the cornea with fluorescein by applying a wetted Fluomet (1 mg Fluorescein Sodium Ophthalmic Strip; Bausch & Lomb, Rochester, NY, USA) into the inferior fornix. The tear film was observed using a biomicroscope with a cobalt blue filter, and the subjects were instructed to blink a few times and then to keep their eyes open as long as possible. The time from the subject’s blink to the first black spot within the tear film was measured. The OSS was assessed using the NEI (National Eye Institute) scale [12]. The cornea was divided into five regions (nasal, central, temporal, inferior, and superior), and a score from 0 (absent) to 3 (severe) was assigned to each region, with a maximum total score of 15. We only analyzed the corneal staining score. All measurements were made by one examiner. For the Schirmer-I test, a standard Schirmer test strip (5 × 40 mm) was placed in the inferior conjunctival sac at the outer third of the inferior lid. The patients were instructed to keep their eyes closed during the test, and the amount of wetting was recorded after 5 min. Using the Schirmer Strip (Color Bar; EagleVision, Memphis, TN, USA) without an anesthetic, results in reflex tearing; hence, it was applied at the end of the tests. The OSDI questionnaire (Allergan, Inc., Irvine, CA, USA) consists of 12 questions related to symptoms within the past week and yields total scores ranging from 0 (least severe) to 100 (most severe). The VAS system consisted of three questionnaires, each with an answer scale ranging from 0 (none) to 10 (very severe) for dryness, foreign body sensation, and pain. Therefore, the total VAS score ranged from 0 to 30. Tear osmolarity was measured using the TearLab Osmolarity System (TearLab Corp., San Diego, CA, USA). Tear samples (50 nL) were collected from the inferior lateral meniscus and analyzed simultaneously. The tear MMP-9 test was performed using a commercially available test kit (InflammaDry; Quidel Corp., San Diego, CA, USA).

### Lipid layer thickness measurement and meibography

LLT was measured using a LipiView^®^ Interferometry (TearScience Inc, Morrisville, NC, USA) by analyzing more than one billion data points of the interferometric image of the tear film. The observed color is related to the LLT and is presented in “interferometric color units” in which 1 interferometric color unit corresponds to approximately 1 nm [13]. The average tear film LLT from all frames was recorded for each study participant. Moreover, images of meibography were obtained using a LipiView^®^ Interferometry from the lower lid and graded using a 0–3 point scale, with 0 as no dropout, 1 as ≤33%, 2 as 34%–66%, and 3 as ≥66% [14].

### Statistical analyses

The data were taken from the eye with a shorter TBUT for statistical analysis. If the TBUT was the same in both eyes, we chose the eye with a thinner lipid layer.

Statistical analyses were performed using SPSS® version 22.0 (IBM Corp., Armonk, NY, USA), and the Mann-Whitney U test was used to compare the dry eye parameters including TEWL and time to reach the plateau between the DED and control groups. The average of five values of TEWL (which means water mass flux at steady state) and time-to-plateau were calculated. Pearson’s correlation test and multiple regression analysis were used to assess the correlations between TEWL and other parameters. All values are expressed as the mean ± standard deviation (SD). A p-value <0.05 was considered statistically significant.

## Results

The demographic characteristics and parameters of interest are presented in Table 1. Data was analyzed from 50 subjects, with a mean age of 35.46 ± 10.38. The mean room temperature was 25.01 ± 1.70°C and the mean relative humidity of the examination room was 35.23 ± 7.49%. Fifty eyes were included in the DED and control groups (25 per group); 12 DED patients and 6 individuals in the control group had MGD.

**Table 1:** Clinical characteristics of the study participants.

Variable	Control group (Mean ± SD or n [%])	DED group (Mean ± SD or n [%])	p-value (Mann-Whitney U test)
Number of eyes	25	25	
Female	21 (84.00%)	19 (76.00%)	
Male	4 (16.00%)	6 (24.00%)	
Age (yrs)	38.12 ± 12.57	38.12 ± 12.57	
TBUT (sec)	7.58 ± 0.94	2.56± 0.85	< 0.001*
Schirmer 1 test (mm)	16.46 ± 10.38	15.94 ± 10.66	0.580
NEI score	0.00 ± 0.00	2.54 ± 1.71	< 0.001*
LLT (nm)	60.33 ± 21.03	78.64 ± 23.18	< 0.001*
Meibography grade	0.22 ± 0.41	0.62 ± 0.77	0.007*
TEWL (g/hm^2/s)	51.85 ± 13.43	52.37 ± 16.38	0.828
Time-to-plateau (sec)	18.30 ± 10.26	40.02 ± 36.47	0.001*
Tear osmolarity (mOsm)	286.27 ± 7.09	287.74 ± 13.80	0.530
OSDI	18.19 ± 20.08	31.22 ± 22.76	0.587
VAS	3.67 ± 4.41	9.48 ± 6.84	0.007*
Positive MMP-9 (n)	8 (32%)	8 (32%)	

DED, dry eye disease; TBUT, tear film break-up time; NEI score, National Eye Institute score; LLT, lipid layer thickness; SD, standard deviation; TEWL, transepidermal water loss; VAS, visual analogue scale; OSDI, ocular surface disease index; MMP-9, tear matrix metallopeptidase 9.

* indicates statistically significant results.

As expected, the TBUT was shorter and the NEI score was higher in the DED group *(*p < 0.001 and p < 0.001, respectively, Mann-Whitney U test). The LLT and meibography grade of the DED group were significantly higher than those of the control group (p < 0.001 and p = 0.007, respectively, Mann-Whitney U test). The time-to-plateau was significantly higher in the DED group (p = 0.001, Mann-Whitney U test). These results indicate that LLT has no effect on tear evaporation, and tear evaporation has no relation to LLT (p = 0.1929, Pearson’s correlation test). To assess the effect of coexisting MGD on TEWL, we performed a subgroup analysis in the two groups (Table 2). There was no statistical difference in TEWL between the four groups. However, the time-to-plateau was significantly different between the DED with MGD group and control group without MGD (42.45 ± 30.94 s, 16.05 ± 6.00 s, respectively; p = 0.018, Mann-Whitney U test).

**Table 2:** Comparison of TEWL and time-to-plateau between the two groups according to the existence of MGD.

	Control group		DED Group		p-value
	MGD (-)	MGD (+)	MGD (-)	MGD (+)	
TEWL (g/hm^2/s)	54.22 ± 13.64	51.78 ± 14.33	52.64 ± 17.93	56.78 ± 20.02	
Time-to-plateau (sec)	16.05 ± 6.00*	22.50 ± 10.65	28.31 ± 39.00	42.45 ± 30.94*	0.018^†^

Data are presented as the mean ± standard deviation. DED, dry eye disease; MGD, meibomian gland dysfunction; TEWL, transepidermal water loss

* indicates statistical significance between two group

^†^ p-value was calculated using the Mann-Whitney U test.

**Fig 1:**
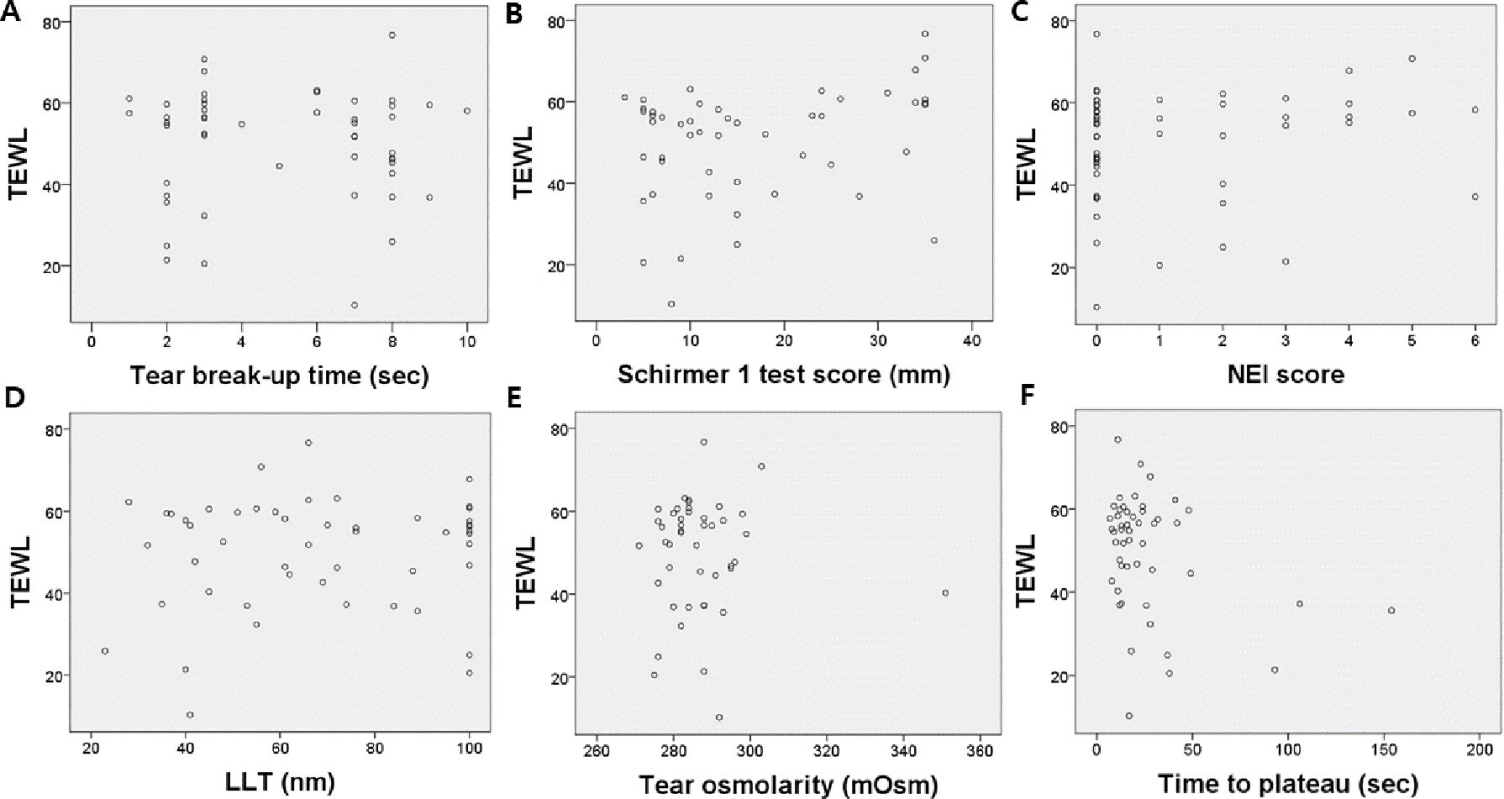
Correlation between TEWL and TBUT (A), Schirmer 1 test scores (B), NEI (National Eye Institute) scores (C), LLT (D), tear osmolarity (E), and time-to-plateau (F) in all participants. (B) Only Schirmer 1 test scores showed a statistically significant correlation with TEWL (*p* = 0.004). TEWL, transepidermal water loss; LLT, lipid layer thickness; TBUT, tear break-up time.

**Fig 2:**
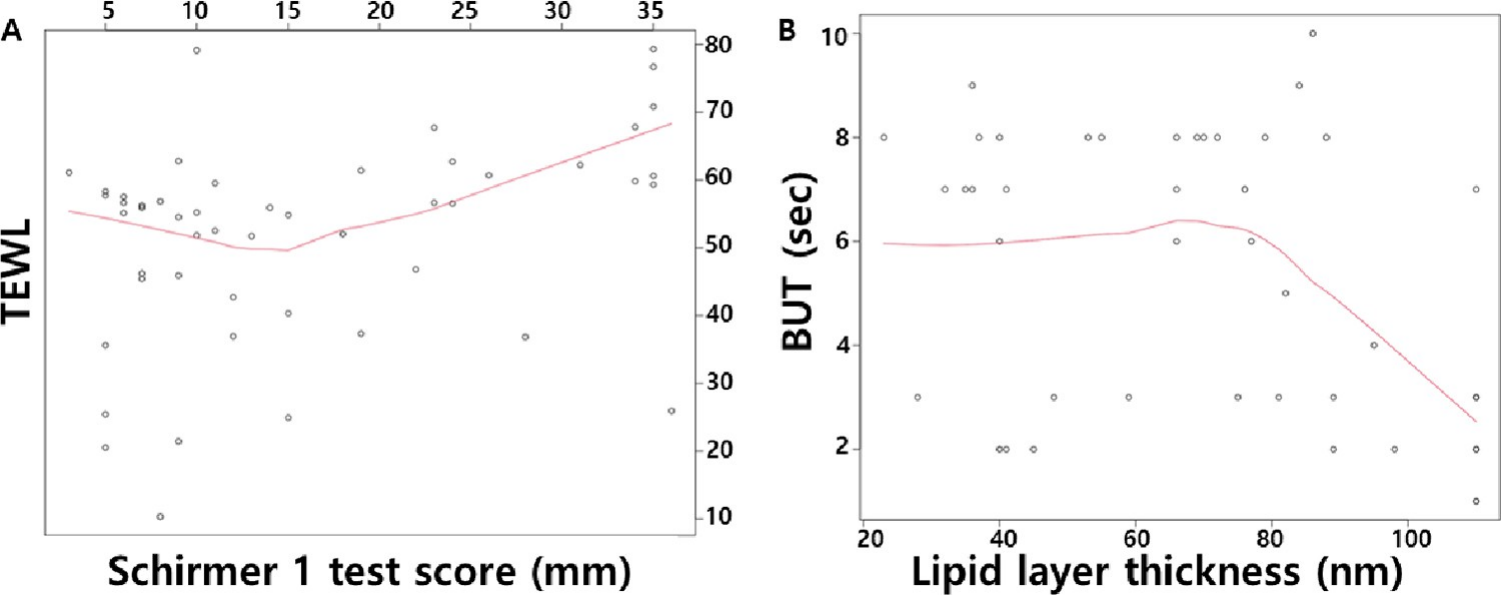
Correlation between TEWL and Schirmer 1 test scores (A), correlation between TBUT tear break-up time) and LLT (B) in all participants. (A) TEWL was correlated with Schirmer 1 test values (*p* = 0.004). (B) TBUT was significantly correlated with LLT (*p* = 0.004) and tended to decrease when LLT was abnormally thick. The red line represents the logically estimated scatterplot smoothing curve. TEWL, transepidermal water loss; LLT, lipid layer thickness; TBUT, tear break-up time.

Furthermore, we analyzed the correlation between TEWL and other DED parameters in the three groups (all participants, the DED group, and the control group) using multiple regression analysis (Fig 1). In all participants, TEWL was correlated with Schirmer 1 test values (p = 0.004) and time-to-plateau (p = 0.023). In patients with short TBUT DED, TEWL tends to increase as tear volume increases (Fig 2A). Time-to-plateau showed no correlation with any other DED parameters in all participants. In the DED group, TEWL was correlated with the Schirmer 1 test (p = 0.001) and NEI score (p = 0.044). Time-to-plateau showed no correlation with any other DED parameters in the DED group. In the control group, TEWL and time-to-plateau were not correlated with other DED parameters. Among the DED parameters, TBUT and NEI were found to be related to LLT (p = 0.004 and p = 0.035, respectively); TBUT tended to decrease when the LLT was abnormally thick (Fig 2B).

## Discussion

In previous studies, LLT was found to be positively correlated with TBUT [15–17], the MGD dropout grade [17], and the Schirmer 1 test score [15]. Increased age, female sex, hypersecretory MGD, and lid margin inflammation were significantly related to increased LLT [18]. In one study, it was reported that there was a correlation between the severity of dry eye symptoms and LLT [19]; however, another study reported that there was no correlation [17]. The results showed that LLT is not significantly correlated with TEWL. This is a somewhat unexpected finding because the lipid layer is thought to be a crucial barrier to excessive tear evaporation in the traditional three-layered tear film model. In vitro validation test is necessary to assess whether the measured TEWL represents the actual tear evaporation rate accurately. LLT showed a significant correlation with TBUT in this study. Local regression implies that TBUT starts to decrease when the lipid layer is thicker than 80 nm. TBUT in hypersecretory MGD was decreased compared to non-MGD or obstructive MGD; however, these differences were marginally significant. These findings suggest that the tear film lipid layer may play a greater role in maintaining the stability of the tear film rather than protecting the aqueous tear evaporation. Nevertheless, we must consider that the discrepancy with previous studies which showed positive correlation between BUT and LLT [15–17] may be due to differences in the subjects’ age, inclusion criteria, and instruments.

While TEWL did not show a significant correlation with LLT, it was positively correlated with the Schirmer-I test value in patients with short TBUT dry eye and healthy controls. Since TEWL was related to the Schirmer 1 test score rather than the LLT, this indicates that it is related to the volume of tears. Contrary to this finding, a previous study showed a negative correlation between TEWL and Schirmer I test values in moderate to severe DED [9]. The reason for showing contradictory results for the Schirmer tear is considered to be a limitation of the traditional Schirmer test itself. According to Kim et al., more accurate results can be derived by using the modified Schirmer’s tear test strips [20]. Additionally, in the same study, TEWL values were significantly different between patients with moderate to severe DED and those in the control group [8]; however, there was no difference between patients with mildly dry eyes and the control group in this study. These contradictory findings can be attributed to the difference in the study population between the two studies. Further, in this study, local regression suggests that TEWL may be negatively correlated with the Schirmer I test values when they are low.

The two main categories of DED are aqueous deficient and evaporative. The current understanding is that these two categories are not mutually exclusive, and there is frequent overlap and co-existence of the two subtypes. As with other DED parameters such as symptoms, tear production, tear film stability, and ocular surface staining, TEWL measurements, in addition, have instrument limitations. In our study, we measured TEWL from the ocular area using a Tewameter TM300 with custom goggles, as reported previously [9]. Our study suggests that water evaporation is positively correlated with Schirmer-1 test. It seems that excessive evaporation may play a role in DED in the setting of low Schirmer wetted length. It is supported by previously reported study that the combination of excessive evaporation and low tear production results in hyperosmolarity and DED [21]. Moreover, the results showed the correlation between LLT and TBUT (Fig 2B). Since TBUT reflects the tear film stability, it can be interpreted that the lipid layer may play a crucial role in the stabilization of tear film. According to a previously published report, tear evaporation rates vary significantly according to different lipid layer patterns. An absent, or non-confluent lipid layer has been determined to be associated with a four-fold increase in evaporation rate [22]. Tomlinson et al. also found that in the presence of a stable, intact lipid layer, tear evaporation was delayed regardless of the lipid layer thickness [22]. The limitation of our study is that only the lipid layer thickness was measured, and the tear lipid layer pattern was not analyzed.

In previous published literature, tear evaporation rate was significantly higher in blepharitics than that in normal [23]. To assess the effect of coexisting MGD, we performed subgroup analyses in the DED and control groups. The time-to-plateau was significantly different between the DED with MGD group and the control group without MGD (42.45 ± 30.94 s, 16.05 ± 6.00 s, respectively; *P* = 0.018, Mann-Whitney U test).

Our study had a few limitations. First, measuring TEWL from the ocular area is not a standard diagnostic method in DED. Even though the equipment is an open-chamber system, there is a chance that the humidity in the goggle may be elevated due to insufficient air circulation. And this can slow down the tear film evaporation rate then actual tear evaporation rate. Therefore, in vitro validation tests are warranted to assess whether the measured TEWL accurately represents the actual tear evaporation rate. Second, as shown in this study, further studies are needed on the effects of various types of dry eye or accompanying factors such as MGD.

## Conclusion

In conclusion, TEWL was not significantly different from the control group in mild short TBUT DED. Further, TEWL was significantly associated with Schirmer 1 test scores but not LLT. These results suggest that the LLT is related to the stability of the tear film and the ocular surface damage (NEI) rather than the amount of evaporation.

## Supporting information

S1 Data (XLSX)
